# Medizinische Kompressionsstrümpfe bei chronischen venösen Erkrankungen und Lymphödem: Wissenschaftliche Evidenz und Ergebnisse einer Patient*innen-Befragung zur Versorgungsqualität

**DOI:** 10.1007/s00105-022-05007-3

**Published:** 2022-06-01

**Authors:** Markus Stücker, Eberhard Rabe

**Affiliations:** 1 Klinik für Dermatologie, Venenzentrum der dermatologischen und gefäßchirurgischen Kliniken, Ruhr-Universität Bochum, Gudrunstr. 56, 44791 Bochum, Deutschland; 2 Praxis für Phlebologie & Dermatologie, Bonn, Deutschland

**Keywords:** Chronische venöse Insuffizienz, Lymphödem, Patientenadhärenz, Patientenaufklärung, Krampfadern, Chronic venous insufficiency, Lymphedema, Patient adherence, Patient education, Varicose veins

## Abstract

**Hintergrund und Ziele:**

Medizinische Kompressionsstrümpfe (MKS) sind bei chronischer venöser Insuffizienz (CVI) aller Stadien indiziert und beim Lymphödem eine unverzichtbare Therapiekomponente; 8 % der deutschen Bevölkerung tragen vom Arzt verordnete MKS, Frauen häufiger als Männer (12 % vs. 5 %) und insbesondere Personen ab 60 Jahren (17 %). Die Adhärenz der Patienten ist relevant für eine erfolgreiche Behandlung mit MKS. Untersucht wurde die Versorgung mit MKS aus Patientensicht.

**Patienten und Methodik:**

Die vorliegende Studie untersuchte 2019 die Versorgungsqualität durch strukturierte Interviews mit 414 repräsentativ ausgewählten Nutzern. Die Erkenntnisse werden vor dem Hintergrund wissenschaftlicher Evidenz zur Wirkung der MKS diskutiert.

**Ergebnisse:**

Venenprobleme sind der häufigste Verordnungsgrund (44 %), gefolgt von Lymphödemen (22 %) bzw. Mehrfachindikationen (27 %). Patienten tragen MKS zumeist täglich und durchschnittlich 11 h/Tag; 89 % der Patienten waren zufrieden bzw. sehr zufrieden mit den MKS und berichteten je nach Indikation ein differenziertes Wirkprofil. Dieses reflektiert die umfangreiche wissenschaftliche Evidenz zur klinischen Wirksamkeit der MKS. Ein wichtiger Faktor für die Patientenadhärenz ist die ärztliche Schulung und Aufklärung.

**Schlussfolgerungen:**

MKS werden von Patienten sehr gut akzeptiert. Bei der Verordnung sollen praktischen Aspekte wie An- und Ausziehen, empfohlene Tragedauer und -häufigkeit sowie der Wirkmechanismus der MKS vermittelt werden.

**Graphic abstract:**

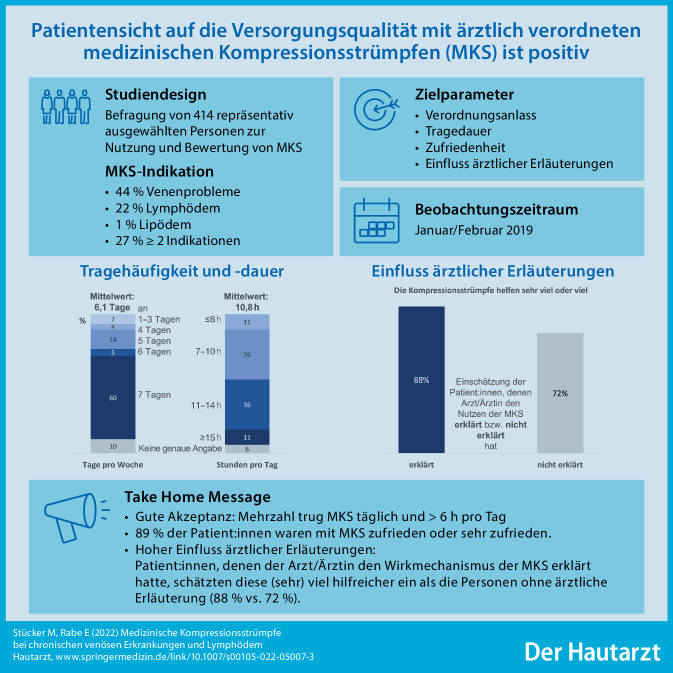

## Abstract (en)

**Background and aims:**

Medical compression stockings (MCS) are indicated for chronic venous insufficiency (CVI) of all stages and are an indispensable component in lymphedema therapy. In all, 8% of the German population has been prescribed MCS by a physician—more women than men (12% vs. 5%), and especially persons aged 60 years and older (17%). Patient adherence is relevant for successful treatment with MCS. We examined MCS use from the patientʼs perspective.

**Patients and methods:**

This study examined the quality of care through structured interviews with 414 representative users in 2019. Findings are discussed taking into consideration scientific evidence on the efficacy of MCS.

**Results:**

Venous conditions are the most common reason for prescription (44%), followed by lymphedema (22%) or multiple indications (27%). Patients generally wear MCS daily and on average 11 h/day. In all, 89% of patients were satisfied or very satisfied with MCS, whereby efficacy was dependent on the indication. This reflects the extensive scientific evidence on the clinical efficacy of MCS. An important factor for patient adherence is physician training and education.

**Conclusion:**

MCS are well accepted by patients. When prescribing them, practical aspects such as donning and doffing, recommended wearing time and frequency, and the mechanism of action of MCS should be clearly communicated.

## Hintergrund

### CVI

Eine Varikose der unteren Extremität findet sich bei etwa 25 % aller Erwachsenen; diese Population überschneidet sich mit der Patientengruppe, die an einer klassischen chronischen venösen Insuffizienz leidet (CVI; ca. 17 %) [1]. Als CVI werden die Stadien C3 bis C6 der sog. CEAP-Klassifikation bezeichnet (CEAP: Clinical, Etiology, Anatomic, Pathophysiology Classification [2]). Sie zeichnen sich durch klinische Befunde wie Beinödeme, Hautveränderungen oder venös induzierte Ulzerationen aus [1, 3–6]. Subjektive Symptome umfassen Schmerz, unspezifische Beschwerden wie Schweregefühl und Schwellung, Krämpfe, Juckreiz, Kribbeln und ruhelose Beine [5, 6]. Pathophysiologisch liegen Veränderungen an den Venen und Venolen mit einem erhöhten venösen Blutdruck zugrunde [6]: Ursachen sind u. a. Varizen, tiefe Venenthrombosen, defekte Venenklappen oder eine unzureichende Muskelpumpe z. B. bei Immobilität [5–8]. Die gestörte Hämodynamik kann Turbulenzen des Blutflusses auslösen, die physiologischen Scherkräfte an der Venenwand reduzieren und inflammatorische und prothrombotische Zytokine ausschütten [1, 3, 5, 9–13]. Unter dem Einfluss der Zytokine öffnen sich kleine Spalten zwischen den Endothelzellen der Venolen und erlauben das Austreten von Blutplasma und die Bildung eines Ödems, einem Frühzeichen der CVI [1, 3, 13–16]. Bei der CVI handelt es sich ohne adäquate Behandlung um eine progrediente Erkrankung, welche die Lebensqualität der Betroffenen negativ beeinträchtigen kann [1, 17].

Die aktuellen Leitlinien der Deutschen Gesellschaft für Phlebologie, die in Zusammenarbeit mit anderen Fachgesellschaften erstellt wurden, sehen in allen Stadien der Varikose und der CVI eine Indikation für die Kompressionstherapie [18, 19]. Durch die Kompression können sich Blutfluss und Scherkräfte in den Venen normalisieren und in der Folge die Ausschüttung der inflammatorischen Zytokine sowie die damit assoziierte Ödembildung.

### Lymphödem

Ein Lymphödem entsteht bei Störung des physiologischen Gleichgewichts zwischen der Nettofiltration von Flüssigkeit durch Blutgefäßwände hindurch ins Interstitium (lymphpflichtige Last) und deren Abtransport über das Lymphdrainagesystem [20]. Bei einem insuffizienten Lymphdrainagesystem vermehrt und verändert sich die interstitielle Gewebeflüssigkeit sukzessive: Unzureichend behandelt, ist das Lymphödem eine progrediente, chronische Erkrankung mit einer Veränderung der extrazellulären Matrix im Sinne einer Fibrose, Sklerose, Entzündung und Vermehrung von Fettgewebe [20–23]. Ein Lymphödem kann infolge einer primären (z. B. genetisch bedingte Dys‑, Hypo- oder Aplasie der Lymphgefäße, Fibrose von Lymphknoten) oder einer sekundären Schädigung des Lymphdrainagesystems auftreten (z. B. Tumoren, Operation, Bestrahlung). Auch eine fortgeschrittene CVI löst ggf. ein sekundäres Lymphödem aus, wenn die lymphatische Drainagekapazität durch vermehrtes Exsudat bei venöser Abflussstörung überfordert wird („high output failure“) [20]. Betroffen von einem Lymphödem sind die initialen Lymphgefäße, das Kollektorsystem, die Lymphstämme und/oder die Lymphknoten [20].

Das primäre Lymphödem befällt nur wenige Personen (Inzidenz bei Geburt ca. 1:6000 [24]). Vom sekundären Lymphödem sind in den Industriestaaten dagegen bis zu 2 % der Bevölkerung betroffen – Frauen 4‑ bis 6‑mal häufiger als Männer [22, 25–27]. Häufigste Ursache ist eine Tumorerkrankung bzw. deren Behandlung (Bestrahlung, Lymphknotenresektion). Adipositas besitzt einen induzierenden und aggravierenden Einfluss [22, 28, 29].

Bei der Behandlung des Lymphödems gilt die Kompressionstherapie laut aktueller Leitlinie der Gesellschaft Deutschsprachiger Lymphologen und der Deutschen Gesellschaft für Lymphologie – erstellt in Zusammenarbeit mit 30 weiteren Fachgesellschaften aus dem deutschsprachigen Raum – als „unverzichtbare Komponente“. Nach einer Ersttherapie (Phase I) mittels mehrlagiger Wechselverbände erfolgt in Phase II die Anwendung von medizinischen Kompressionsstrümpfen [22, 30].

### Medizinische Kompressionsstrümpfe

Die Leitlinie zur medizinischen Kompressionstherapie der Extremitäten, die im Dezember 2018 überarbeitet und 2019 veröffentlicht wurde, bezeichnet die Therapie mit medizinischen Kompressionsstrümpfen (MKS) als unverzichtbar bei der Behandlung phlebologischer und lymphologischer Erkrankungen der Extremitäten [18]. Der MKS wird als „strumpfförmiges, elastisches Gestrick“ beschrieben, das durch seine elastischen Eigenschaften den venösen und lymphatischen Abstrom und die venöse Pumpfunktion verbessert. MKS werden sowohl zur Prävention als auch zur längerfristigen Therapie und Erhaltung eines Therapieerfolgs eingesetzt. Sie sind flachgestrickt mit Naht und als rundgestrickte MKS ohne Naht erhältlich. Die Variante mit Naht lässt eine bessere Anpassung an die Form der Beine zu, wenn der Umfang stark variiert, und vermeidet bei Bewegung das Auftreten von Druckspitzen durch Einschnürungen insbesondere bei Hautfaltenbildungen z. B. bei fortgeschrittenen Stadien des Lymphödems und des Phlebödems.

Die vorliegende Studie untersuchte die Versorgungsqualität mit MKS aus Sicht der betroffenen Patientengruppen, da die Adhärenz der Patienten zur Kompressionstherapie sich als relevant für eine erfolgreiche Behandlung sowohl bei phlebologischer als auch bei lymphologischer Indikation herausgestellt hat [18, 19, 22, 31].

## Ziele der Untersuchung und Methodik

Untersucht wurde im Auftrag der European Manufacturers Federation for Compression Therapy and Orthopaedic Devices (eurocom e. V.) die Fragestellung, ob und wie Patienten ärztlich verordnete MKS nutzen und bewerten. Die Bevölkerungsbefragung erfolgte mehrstufig durch das Institut für Demoskopie Allensbach. Im ersten Schritt wurde in einer repräsentativen Bevölkerungsbefragung ermittelt, wer medizinische Hilfsmittel verwendet, um die Grundverteilung in der Bevölkerung festzustellen. In einem zweiten Schritt suchten die Interviewer für die Befragung Personen, die MKS nutzen. Auf der Basis der Bevölkerungsbefragung konnte die erforderliche Verteilung bezüglich der Merkmale Geschlecht (2 Gruppen), Alter (3 Gruppen) und Region (7 Gruppen) berücksichtigt und die Repräsentativität der Ergebnisse erreicht werden; 414 Personen wurden im Januar und Februar 2019 von Mitarbeitern des Instituts für Demoskopie Allensbach persönlich befragt.

Die befragten Personen wurden vom Interviewer über die Erhebung und Verarbeitung ihrer Daten aufgeklärt und erklärten dazu mündlich ihr Einverständnis. Das Institut für Demoskopie Allensbach anonymisierte die Daten vor Weitergabe an eurocom durch Aggregation und stellte damit sicher, dass sich die Daten keiner bestimmten Person mehr zuordnen ließen. Die Befragung erfüllte daher nicht die Definition eines Forschungsvorhabens nach § 15 der ärztlichen Musterberufsordnung, und die Beratung durch eine medizinische Ethikkommission war nicht erforderlich.

Berichtet werden hier die Ergebnisse der Interviews mit 414 Nutzern von ärztlich verordneten MKS. Personen, die nicht vom Arzt verordnete Stützstrümpfe verwendeten oder nach Klinikaufenthalt Thromboseprophylaxestrümpfe weiter nutzten, wurden nicht in die Stichprobe aufgenommen.

Erhobene Daten der Befragung waren insbesondere Verordnungsanlass der MKS (Frage der Interviewer nach „Venenproblemen, Krampfadern, Varizen“ und/oder „Lymphödem, Wasser in den Beinen“ und/oder „Lipödem, Fettablagerungen – häufig seitlich an den Hüften oder Oberschenkeln“), Tragedauer und -frequenz, Zufriedenheit mit MKS, Wirkprofil, Mobilität, Lebensqualität (Frage der Interviewer „Die Kompressionsstrümpfe haben mir geholfen, ein Stück Lebensqualität zurückzugewinnen“), Unzufriedenheit mit MKS, subjektiv wichtige Aspekte und Informationen zu MKS.

In dieser Arbeit wird anhand eines selektiven Reviews von Leitlinien und Übersichtsarbeiten der Frage nachgegangen, ob das Wirkprofil der MKS aus Patientensicht einer rein subjektiven Wahrnehmung entspringt oder ob hinreichende wissenschaftliche Evidenz zur Wirksamkeit von MKS besteht.

## Ergebnisse

### Allensbach-Umfrage

**Figure 1:**
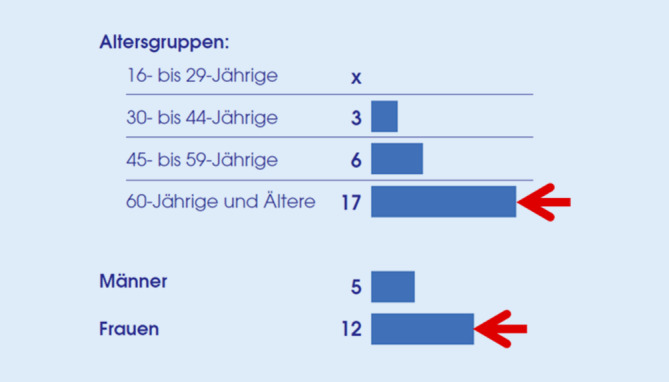

Eine Strukturanalyse der Population im Rahmen der oben genannten Repräsentativbefragung ergab, dass insgesamt 8 % der deutschen Bevölkerung vom Arzt verordnete MKS tragen; dies entspricht etwa 5 Mio. Personen. Frauen verwenden MKS mehr als doppelt so oft wie Männer (12 % vs. 5 %). Von den über 60-Jährigen verwenden 17 % MKS, in der Population von 30 bis 44 Jahren 3 % (Abb. 1).

Anlass für die Verordnung von Kompressionsstrümpfen waren bei knapp der Hälfte der Patienten (44 %) ausschließlich Venenprobleme gemäß obiger Fragestellung, bei 22 % ausschließlich ein Lymphödem und bei 1 % ausschließlich ein Lipödem. Bei 27 % der Nutzer von MKS lagen mindestens 2 verschiedene Indikationen vor. Personen mit Normalgewicht trugen MKS weit überwiegend (59 %) wegen Venenproblemen, bei adipösen Patienten (BMI ≥ 30 kg/m^2^) verteilten sich die Indikationen etwa gleichmäßig auf Venenprobleme allein, Lymphödem allein und eine Kombination mehrerer Anlässe.

**Figure 2:**
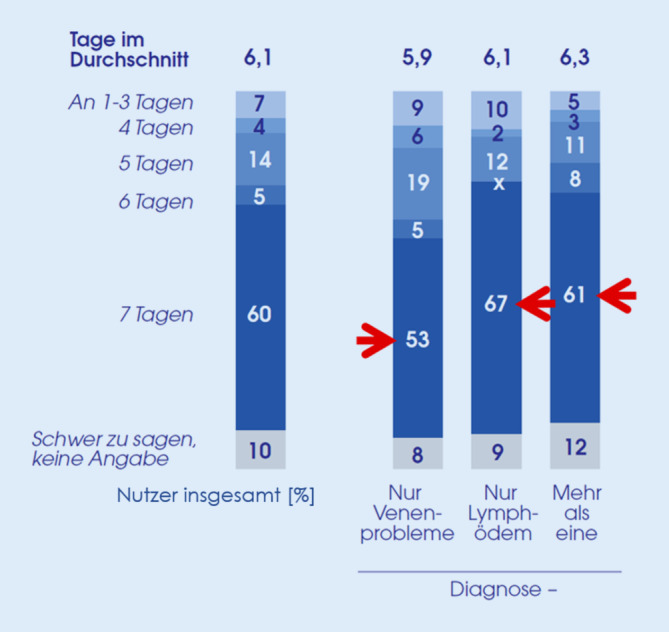

Die durchschnittliche Nutzungsdauer der MKS lag bei 7,2 Jahren mit nur geringen Unterschieden zwischen den Altersgruppen. Bei Frauen war die durchschnittliche Nutzungsdauer länger als bei Männern (7,7 vs. 5,4 Jahre). Patienten mit mehreren Indikationen trugen MKS seit längerer Zeit als Patienten mit ausschließlich Venenproblemen oder Lymphödem (8,3 vs. 7,1 vs. 6,1 Jahr); 60 % der Patienten tragen ihre MKS täglich; am höchsten lag dieser Anteil bei Patienten mit einem Lymphödem (67 %; Abb. 2).

Die durchschnittliche Tragedauer pro Tag lag bei knapp 11 h mit nur geringen Unterschieden zwischen den Indikationen. Neun bzw. 2 % der befragten Patienten berichteten, ihre MKS zwischen 15 und 19 h bzw. zwischen 20 und 24 h pro Tag zu tragen; beide Kategorien implizieren ein Tagen der MKS auch bei Nacht. Diese Anteile lagen bei Männern höher als bei Frauen (insgesamt 17 % vs. 8 %) und bei älteren Patienten tendenziell höher als in der Altersgruppe unter 60 Jahren (12–13 % vs. 7 %). Anders als erwartet, fanden sich bei den Patienten mit Lymphödem keine Hinweise auf ein häufigeres nächtliches Tragen der MKS. Es zeigte sich ein Zusammenhang zwischen der Häufigkeit des Tragens von MKS und der Tragedauer: Tägliche Anwender kamen auf eine Tragedauer von 12 h, Patienten mit seltenerer Anwendung auf 9 h.

**Figure 3:**
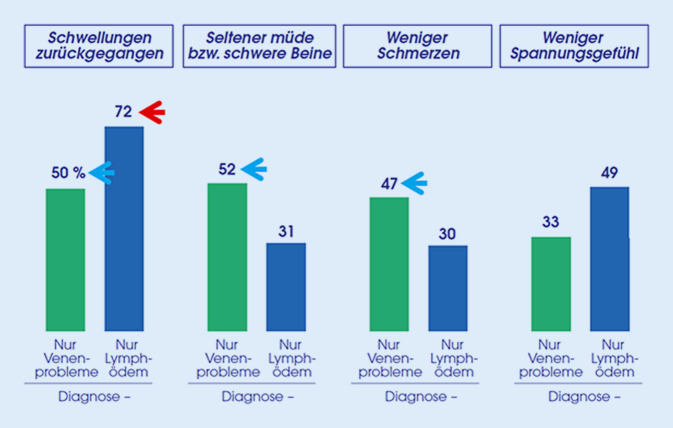

Je nach Diagnose zeigte das Wirkprofil differenzierte Effekte. Patienten mit *Lymphödem* berichteten insbesondere, dass ihre Schwellungen zurückgegangen seien (72 %), wohingegen die Verbesserung von Schmerzen bzw. müden und schweren Beinen (je ca. 30 %) weniger im Vordergrund stand. Bei Patienten mit *Venenproblemen* zeigte sich dagegen eine weitgehend gleichmäßige Verbesserung dieser 3 Symptome: eine Reduktion von Schwellungen, müden/schweren Beinen und/oder von Schmerzen berichtete etwa jeder zweite Patient (Abb. 3; Mehrfachnennung möglich). Zugleich berichteten vergleichbar viele Patienten, dass sich die Symptome verschlechtern, wenn die MKS zwischendurch nicht getragen werden.

**Figure 4:**
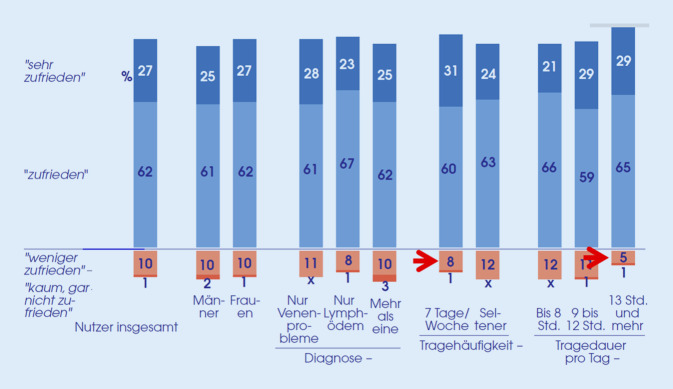

Es waren 89 % der Patienten zufrieden oder sehr zufrieden mit ihren MKS (Abb. 4). Unterschiede zwischen Patientinnen und Patienten sowie zwischen den Indikationen waren gering, jedoch waren Patienten mit hoher Tragehäufigkeit zufriedener als Patienten, die ihre MKS seltener trugen (91 % vs. 87 % zufrieden oder sehr zufrieden). Dies galt ebenso für die Tragedauer pro Tag: Patienten mit langer Tragedauer ab 13 h pro Tag waren zufriedener als Patienten, die ihre MKS kürzer trugen (94 % vs. 88 % zufrieden oder sehr zufrieden).

**Figure 5:**
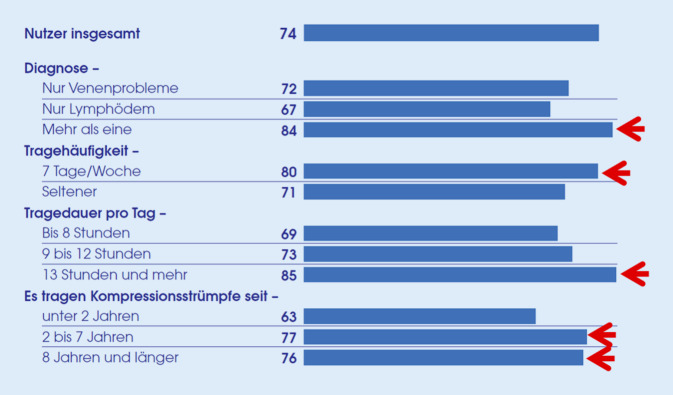

Insgesamt gaben 85 % der Patienten an, dass es ihnen viel oder sehr viel helfe, ihre MKS zu tragen, 61 % fühlten sich dadurch mobiler; 74 % der Patienten gaben an, dass die MKS ihnen geholfen hätten, ein Stück Lebensqualität zurückzugewinnen. Insbesondere Patienten mit mehr als einer Indikation für MKS, Patienten mit täglicher Anwendung, mit langer Tragedauer und langer Erfahrung mit MKS berichteten einen Gewinn an Lebensqualität (Abb. 5).

Lediglich 11 % der Patienten waren weniger oder nicht zufrieden mit ihren MKS. Als Hauptgründe nannten sie einen schlechten Tragekomfort (7 %) sowie Probleme beim An- und Ausziehen (5 %). Dementsprechend war es 82 % bzw. 81 % der Patienten wichtig, dass sie ihre MKS gut an- und ausziehen können bzw. dass diese beim Tragen keine Hautprobleme verursachen. Eine Anziehhilfe besaßen 49 % der Patienten, 25 % wurde sie ärztlich verordnet. Hautpflegemittel zur Verhinderung von Hautproblemen verwendeten 64 % der Patienten, darunter deutlich mehr Frauen als Männer (69 % vs. 51 %). Mehr als jedem zehnten Patienten war die Verfügbarkeit von Hautpflegemitteln nicht bekannt.

**Figure 6:**
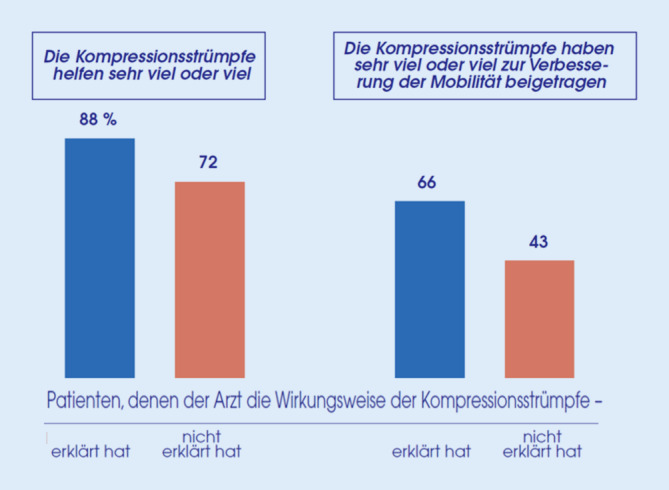

Die Interviews zeigten eine deutliche Auswirkung der ärztlichen Erläuterungen auf die Perzeption der MKS: Patienten, denen ihr Arzt den Wirkmechanismus der MKS erklärt hatte, waren eher der Meinung, dass MKS sehr viel oder viel helfen, als diejenigen, die derartige Erklärungen nicht bekommen hatten (88 % vs. 72 %). Ein analoger Zusammenhang mit den ärztlichen Erläuterungen fand sich beim Eindruck der Patienten, dass ihre MKS sehr viel oder viel zur Verbesserung der Mobilität beigetragen haben (66 % vs. 43 %; Abb. 6).

Zusammenfassend fand die repräsentative Umfrage bei 414 Patienten, die MKS tragen, eine große Zufriedenheit mit dieser Therapie. Die Befragung zeigte, dass Patienten umso zufriedener mit ihren MKS waren, je besser sie im Vorfeld durch den Arzt über die Wirkung und Wirkungsweise der MKS aufgeklärt wurden. Weiterhin fand sich ein deutlicher Zusammenhang zwischen der Zufriedenheit mit der Therapie und regelmäßigem und langem Tragen der MKS. Methodologisch bedingt, kann diese Befragung weder eine Kausalität dieses Zusammenhangs noch eine etwaige Richtung belegen. – Tragen Patienten, die mit MKS und Symptomlinderung zufrieden sind, die MKS regelmäßig und lange, um diesen Therapieerfolg nicht zu gefährden, oder begründet regelmäßiges und langes Tragen der MKS einen guten Therapieerfolg?

### Wissenschaftliche Evidenz

#### CVI

Die Clinical Practice Guidelines der European Society for Vascular Surgery (ESVS) zum Management der CVI sieht auf der Basis größerer nicht randomisierter Studien, eines Cochrane-Reviews und einer randomisierten Studie Evidenz der zweithöchsten Klasse B, dass MKS klinische Symptome der CVI (Stadium C0–C4) sowie die Lebensqualität verbessern, und spricht eine Empfehlung der höchsten Klasse I aus [32]. Positive Evidenz für die Kompression findet sich auch in den höheren Stadien der CVI (C5 und C6) bezüglich der Heilung von Hautulzerationen und der Verhinderung deren Wiederauftretens. Dabei sollte jeweils ein möglichst hoher Anpressdruck angestrebt werden.

Ein Consensus-Statement aus dem Jahr 2016 zur Therapie der chronischen Veneninsuffizienz unterstützt diese Schlussfolgerungen und gibt – für alle Stadien der CVI – auf der Basis umfassender Evidenz der Klasse A eine Empfehlung der höchsten Klasse 1 für die Kompressionstherapie [13]. Das Consensus-Statement unterstützt die Empfehlung der ESVS zu einem hohen Anpressdruck bei Patienten mit Hautulzerationen. Weiterhin betonen die Autoren die Bedeutung einer guten Compliance der Patienten, die bei MKS höher liegt als bei anderen Kompressionssystemen und z. B. durch die Auswahl von Anziehhilfen gesteigert werden kann.

Ein aktuelles Consensus-Statement zur Anwendung von MKS bei venösen und lymphatischen Erkrankungen aus dem Jahr 2018 kommt zu vergleichbaren Schlussfolgerungen wie die ESVS und empfiehlt MKS zur Linderung von Symptomen sowie zur Verbesserung der Lebensqualität und zur Beseitigung von Schwellungen/Ödemen der Beine [33]. Die Autoren weisen darauf hin, dass auch MKS mit niedrigem Anpressdruck (10–20 mm Hg) Beschwerden lindern und die Compliance der Patienten mit der Therapie verbessern können. Auf der Basis der vorliegenden Studiendaten sei es wichtig, MKS mit individuell an den Schweregrad der CVI angepasstem Anpressdruck zu verordnen.

Die ESVS-Guidelines und die beiden Consensus-Statements flossen beide in die bereits erwähnten Leitlinien zur Diagnostik und Therapie der Varikose und zur Kompressionstherapie der Deutschen Gesellschaft für Phlebologie und anderer Fachgesellschaften ein [18, 19]. Sie schließen auf der Basis multipler Einzelstudien, von Übersichtsarbeiten und mehrerer Cochrane-Reviews [31, 34, 35], dass die Kompressionstherapie venöse Ödeme reduziert. Das Ausmaß der Wirkung ist vom Ruheanpressdruck und der Materialfestigkeit der MKS abhängig: je höher, desto ausgeprägter ist der Effekt. Diese Abhängigkeit von der „Dosis“ gilt als Nachweis einer Wirksamkeit. MKS können zur Reduktion eines bestehenden Ödems eingesetzt werden sowie zur Prävention des Erstauftretens bei frühen Stadien der CVI bzw. des Neuauftretens nach Entstauung. Die umfangreichsten Daten zur Kompressionstherapie und auch die höchste Evidenz zu ihrer Wirksamkeit liegen für die höheren Stadien der CVI vor (C5 und C6), d. h. für die Stadien mit abgeheiltem bzw. floridem Ulkus. Zur Ulkusbehandlung oder Prävention werden Ulkuskompressionsstrümpfe (UKS) verwendet, eine zweiteilige Spezialform der MKS mit Unter- und Oberstrumpf, die nach einer verwandten Norm gefertigt werden.

Weiterhin lindert die Kompressionstherapie mit MKS auch klinische Symptome der CVI wie Schweregefühl, Parästhesien und Neigung zu Schwellungen, z. B. nach langem Sitzen oder Stehen. Zur Reduktion der Symptome genügt häufig ein niedriger Ruheanpressdruck (bis ca. 20 mm Hg) [18, 19].

#### Lymphödem

Das erwähnte Consensus-Statement von Rabe et al. zur Anwendung von MKS bewertet auch Studiendaten zur Anwendung der Kompressionstherapie bei lymphatischen Erkrankungen [33]. Auch die Leitlinie zur Medizinischen Kompressionstherapie widmet sich in einem kurzen Kapitel den klinischen Daten [18]. Die Behandlung des Lymphödems folgt einer multimodalen Kompressionstherapie („Komplexe Physikalische Entstauungstherapie“), MKS können in allen Phasen eingesetzt werden. Zu Beginn der Behandlung wird stets auch eine manuelle Lymphdrainage empfohlen. Eine Empfehlung höchster Evidenzklasse (1A) für MKS besteht für die Langzeiterhaltungstherapie bei Lymphödem. Dabei sollte der höchste Anpressdruck verordnet werden, den die Patienten tolerieren (20–60 mm Hg).

Die Leitlinie zur Diagnostik und Therapie der Lymphödeme beschreibt die Wirkungen der Kompressionstherapie und betont, dass in der Erhaltungsphase nach initialer Entstauung nicht nur eine Ödemreduktion, sondern auch eine Verbesserung der Gewebebefunde möglich ist [22]. Die Autoren weisen darauf hin, dass eine Anfertigung der MKS nach Maß erforderlich ist und dass zum Erreichen eines ausreichenden Anpressdrucks ggf. auch 2 MKS übereinander getragen werden müssen.

Im Jahr 2019 erschien ein aktueller Review zur Prävention des postthrombotischen Syndroms (PTS) mittels MKS. PTS kann als Form einer sekundären chronischen venösen Erkrankung betrachtet werden [36]. Avila et al. analysierten 4 von ursprünglich 831 recherchierten Studien, die den Einschlusskriterien der Metaanalyse genügten. Die Studien verglichen die Anwendung von MKS mit Placebostrümpfen (1 Studie) oder keiner Behandlung (3 Studien) innerhalb eines Monats nach der Diagnose einer tiefen Venenthrombose. Der Zeitraum der Nachbeobachtung bis zum Auftreten oder Nichtauftreten eines PTS lag zwischen 6 und 52 Monaten; die Länge der Nachbeobachtung wurde in der Auswertung berücksichtigt. Die Studien zeigten bei merklicher Heterogenität numerische Vorteile für MKS. Das Risiko („odds ratio“), ein PTS jeglichen Schweregrads zu erleiden, reduzierte sich durch MKS auf 0,57 im Vergleich zu den Kontrollgruppen (95 %-Vertrauensintervall 0,21–1,20), das Risiko, ein schwerwiegendes PTS (vs. kein, leichtes oder mittelschweres PTS) zu erleiden, auf 0,79 (0,31–1,67). Die Autoren schließen, dass MKS bei der Verhinderung von PTS nach tiefer Venenthrombose eine Rolle spielen können. Sie unterstützen damit die Schlussfolgerungen eines früheren Cochrane Reviews [37].

## Diskussion

MKS sind umfassend klinisch untersucht. Eine MEDLINE-Recherche im Februar 2022 fand zu den Stichworten „compression stockings“ und „clinical trial“ über 700 Literaturstellen. Die wissenschaftliche Evidenz wurde, wie oben dargestellt, in mehreren aktuellen Leitlinien, Übersichtsarbeiten und systematischen Metaanalysen zusammengetragen, u. a. in Cochrane-Reviews. Die klinische Datenlage zeigt belastbare Evidenz für eine klinische Wirksamkeit von MKS bei Venenerkrankungen und beim Lymphödem. Insofern erscheint es plausibel, dass die Verbesserung von Symptomen wie Schwellungen, müden/schweren Beinen und Schmerzen, die Patienten in der Allensbach-Umfrage berichteten, nicht auf einem Placebo- oder Regression-to-the-mean-Effekt beruhen.

Der Anwendung von MKS stehen dennoch Hürden entgegen. Häufig wird davon ausgegangen, dass Patienten Kompressionsstrümpfe skeptisch betrachten und ihre Adhärenz zu ihnen gering ist. Auch Consensus-Statements und Übersichtsarbeiten berücksichtigen in ihren Therapieempfehlungen – neben tatsächlichen Kontraindikationen gegen die Kompressionstherapie wie Hautkrankheiten, peripheren Durchblutungsstörungen oder eingeschränkter Beweglichkeit, die beim An- und Ausziehen von MKS behindert – eine vermutete oder tatsächlich unzureichende Adhärenz der Patienten und benennen Adhärenz-freundliche Alternativen wie orale Arzneimittel: Da Venenerkrankungen ohne Therapie generell progredient verlaufen [1, 17], ist bei konservativer, d. h. nichtchirurgischer Therapieindikation die ausreichende Adhärenz zu einer Langzeittherapie essenziell [1, 3, 13]. So führt bei CVI-bezogenen Ulzera eine höhere Adhärenz zu geringeren Rezidivraten [38].

In der Tat berichten Studien, dass die Adhärenz zu MKS bei ca. 50 % und geringer lag [39–41]. Allerdings ist die Datenlage zur tatsächlichen Adhärenz durch eine Vielzahl verwendeter Skalen, Definitionen und Cut-offs unübersichtlich. Ein Review von Bar et al. berichtet Raten zwischen 20 und 95 %, wobei in den eingeschlossenen Studien teilweise Kriterien wie das Einlösen des MKS-Rezepts und die Durchführung empfohlener Übungen berücksichtigt wurden, die über übliche Adhärenzkriterien hinausgehen [42].

Die aktuelle Allensbach-Umfrage und eine frühere Umfrage zum selben Thema aus dem Jahr 2014 liegen im oberen Mittelfeld der berichteten Adhärenzdaten: Im Durchschnitt trugen Patienten ihre verordneten MKS an mehr als 6 Tagen pro Woche. Wurden Patienten als adhärent definiert, die eine Tragehäufigkeit von 6 oder 7 Tagen pro Woche berichten (im Vergleich zu den Studien im oben referierten Review eine eher strenge Definition), lag die Adhärenz bei mindestens 65 %. Dabei sollte berücksichtigt werden, dass Patienten bei Interviews und in Studien ihre Adhärenz im Sinne einer sozialen Erwünschtheit ggf. zu positiv darstellen; dies fällt bei einem Abgleich mit Sensordaten auf, die in Studien-Settings in MKS integriert wurden [42, 43].

Gängige Vorurteile und Daten zur Adhärenz unterschätzen jedoch die allgemeine Zufriedenheit der Patienten mit MKS: Kritikpunkte der Patienten fand die Umfrage lediglich zu isolierten Aspekten, die generell als lösbar betrachtet werden. So berichteten 5 % der befragten Patienten Schwierigkeiten beim An- und Ausziehen der Strümpfe. Dies ist auch ein wichtiger psychologischer Aspekt: Patienten empfinden es wichtig für ihre Autonomie, MKS anlegen und ausziehen zu können, ohne auf fremde Hilfe angewiesen zu sein [44]. Dennoch besitzt nur etwa die Hälfte der Patienten eine An- und Ausziehhilfe, wiederum nur jede zweite davon wurde den Patienten ärztlich verordnet. Dabei können An- und Ausziehhilfen bei gegebener Indikation zulasten der Krankenkassen verordnet werden, wie dies auch eine Empfehlung aus der aktuellen Leitlinie zur medizinischen Kompressionstherapie darstellt [18]: „Bei eingeschränkter Beweglichkeit und Problemen beim An- und Ausziehen des MKS sollten geeignete An- und Ausziehhilfen verordnet werden.“

Zu den verordnungsrelevanten Indikationen für An- und Ausziehhilfen zählen

- Lähmungen,
- altersbedingte Kraftminderungen,
- Arthrose/Rheuma,
- Adipositas permagna,
- weitgehende Wirbelsäulen‑/Hüft‑/Knieversteifungen,
- degenerative Erkrankungen der Hände/im Handbereich,
- Folge von Verletzungen/Amputationen.

Der wichtigste isolierte Kritikpunkt der befragten Patienten an ihren MKS betraf den Tragekomfort (7 %). Auch hier besteht Optimierungspotenzial, z. B. durch die Wahl einer auf das individuelle Extremitätenprofil zugeschnittenen Form, um Druckstellen und Einschneiden zu vermeiden. Auch das Thema Hautpflege ist im Rahmen des Tragekomforts relevant, um Infektionen, Irritationen und Trockenheit der Haut zu vermeiden. Die Existenz entsprechender Pflegemittel war in der Umfrage immerhin 10 % der MKS-Trägerinnen und -Träger nicht bekannt. Die Leitlinie zur Diagnostik und Therapie des Lymphödems äußert sich ausführlich zu diesem Thema und empfiehlt außer einer pH-neutralen Reinigung eine tägliche Pflege, um „Sekundärinfektionen, z. B. durch Rhagaden, zu vermeiden und die Barrierefunktion der Haut zu erhalten“. Die Verträglichkeit des Pflegeprodukts mit den Materialien des MKS ist heute in der Regel kein Problem mehr, da moderne MKS ihre Elastizität durch synthetische Fasern und nur noch in Ausnahmefällen durch den empfindlichen Latex gewinnen. Gegebenenfalls können auch MKS mit integriertem Hautschutz in Betracht gezogen werden, die über die Lebensdauer des MKS lipophile, hautpflegende Substanzen aus dem Gewebe freisetzen [45]. Als Teil der Hautpflege sollte aus Hygienegründen ggf. ein Zweitpaar der MKS verordnet werden, um während des Waschens und Trocknens die Compliance nicht zu gefährden. Generell sollten MKS nach ca. 6 Monaten erneuert werden, um ein Absinken des Anpressdrucks durch Nutzung und Reinigung zu vermeiden.

Wesentliches Element des Tragekomforts ist der Anpressdruck der MKS. Der Einfluss des Anpressdrucks auf die Compliance ist gut untersucht: Diese ist höher bei niedrigerer Klasse des MKS, d. h. bei niedrigerem Anpressdruck (z. B. [34, 40, 46, 47]). Hier existiert ein Interessenkonflikt, denn wie erwähnt steigt die Wirksamkeit von MKS generell mit dem Anpressdruck. Im Zweifelsfall ist jedoch ein täglich getragener MKS mit mittlerem Anpressdruck wirksamer als ein nicht getragener MKS einer höheren Klasse. Für die Wahl des geeigneten MKS ist auch eine Erkenntnis aus dem stationären Bereich interessant, nach der knielange MKS besser akzeptiert werden als hüftlange [48, 49]. Aus dem ambulanten Umfeld sind Erkenntnisse publiziert, dass knielange MKS mit niedrigerem Anpressdruck (18–21 mm Hg) bei signifikant besserem Tragekomfort die Symptome Brennen und Schwellung vergleichbar verbessern wie MKS mit höherem Anpressdruck (23–32 mm Hg); allenfalls bei der Reduktion von Schmerzen zeigten sie eine etwas geringere Wirkung [47]. Darüber hinaus berichten die Autoren, dass MKS mit höherem Anpressdruck bei Patienten mit orthopädischen Fußdeformitäten zu Druckstellen und Einschränkungen der Beweglichkeit führten.

Wesentliche Aspekte zum Tragekomfort von MKS sind:

- passende Kompressionsklasse (Anpressdruck),
- Auswahl der passenden Form,
- Hautpflege, extern oder integriert,
- Zweitpaar, Erneuerung nach 6 Monaten.

Abschließend soll die zentrale Rolle unterstrichen werden, die der Patientenaufklärung und Schulung bei der Therapie mit MKS zukommt. Die Allensbach-Umfrage fand eine deutlich höhere Therapiezufriedenheit bei Patienten, denen in der ärztlichen Praxis die Wirkungsweise der MKS erklärt wurde. Andere Autoren zeigten, dass eine Schulung der Patienten sowohl das Verständnis für Erkrankung und Therapie als auch die Adhärenz zu MKS deutlich erhöhen kann [41, 44, 50]. Eine französische Arbeitsgruppe fand eine um rund ein Drittel längere tägliche Tragedauer bei Patientinnen, die mit Schulung, Empfehlungen und wöchentlichen Erinnerungen per SMS betreut wurden, verglichen mit der Gruppe, die nur absolut notwendige Erklärungen erhielten. Die Compliance war um rund 44 % höher (70 % vs. 48,5 %). Die Tragedauer wurde über ein objektives Verfahren bestimmt, die Messung der Hauttemperatur unter den MKS [39].

Die Schulung der Patienten sollte neben praktischen Aspekten wie dem An- und Ausziehen und der empfohlenen Tragedauer und -häufigkeit explizit auch auf eine patientenverständliche Weise medizinische Informationen vermitteln [39, 44, 50]: Wirkung und Wirkmechanismus der MKS auf Basis der Pathophysiologie der jeweils therapierten Erkrankung, zu erwartende Effekte und deren Ausmaß, Therapiezufriedenheit von Patienten, die MKS tragen, und Kongruenz mit der Wirksamkeit in klinischen Studien. Patienten sollen ihre MKS als ein aktives Therapiesystem einordnen und adoptieren, eine standardisierte Schulung unter Zuhilfenahme grafischer Schaubilder oder Videoanimationen wird empfohlen. Dies ist im Sinne eines *Patient Empowerment* zu verstehen, das den Patienten die Kontrolle über ihre Erkrankung zurückgibt und eine passive Rolle vermeidet.
